# Apo and ligand-bound high resolution Cryo-EM structures of the human Kv3.1 channel reveal a novel binding site for positive modulators

**DOI:** 10.1093/pnasnexus/pgac083

**Published:** 2022-06-16

**Authors:** Mathieu Botte, Sophie Huber, Denis Bucher, Julie K Klint, David Rodríguez, Lena Tagmose, Mohamed Chami, Robert Cheng, Michael Hennig, Wassim Abdul Rahman

**Affiliations:** leadXpro AG, PARK InnovAARE, 5234 Villigen, Switzerland; leadXpro AG, PARK InnovAARE, 5234 Villigen, Switzerland; leadXpro AG, PARK InnovAARE, 5234 Villigen, Switzerland; H. Lundbeck A/S, Ottiliavej 9, 2500 Valby, Denmark; H. Lundbeck A/S, Ottiliavej 9, 2500 Valby, Denmark; H. Lundbeck A/S, Ottiliavej 9, 2500 Valby, Denmark; BioEM laboratory, Biozentrum, University of Basel, Spitalstrasse 41, 4056, Basel, Switzerland; leadXpro AG, PARK InnovAARE, 5234 Villigen, Switzerland; leadXpro AG, PARK InnovAARE, 5234 Villigen, Switzerland; leadXpro AG, PARK InnovAARE, 5234 Villigen, Switzerland

**Keywords:** Kv3.1, potassium channels and modulator

## Abstract

Kv3 ion-channels constitute a class of functionally distinct voltage-gated ion channels characterized by their ability to fire at a high frequency. Several disease relevant mutants, together with biological data, suggest the importance of this class of ion channels as drug targets for CNS disorders, and several drug discovery efforts have been reported. Despite the increasing interest for this class of ion channels, no structure of a Kv3 channel has been reported yet. We have determined the cryo-EM structure of Kv3.1 at 2.6 Å resolution using full-length wild type protein. When compared to known structures for potassium channels from other classes, a novel domain organization is observed with the cytoplasmic T1 domain, containing a well-resolved Zinc site and displaying a rotation by 35°. This suggests a distinct cytoplasmic regulation mechanism for the Kv3.1 channel. A high resolution structure was obtained for Kv3.1 in complex with a novel positive modulator Lu AG00563. The structure reveals a novel ligand binding site for the Kv class of ion channels located between the voltage sensory domain and the channel pore, a region which constitutes a hotspot for disease causing mutations. The discovery of a novel binding site for a positive modulator of a voltage-gated potassium channel could shed light on the mechanism of action for these small molecule potentiators. This finding could enable structure-based drug design on these targets with high therapeutic potential for the treatment of multiple CNS disorders.

Significance Statement Kv potassium channels modulate the electrical activity of cells by opening and closing, following changes in membrane potential. The Kv3 family members, which are characterized by their capacity to open at depolarized membrane potential, are responsible for high frequency firing. Kv3 channels are important therapeutic targets particularly for seizure treatment. We present the structure of Kv3.1, which reveals a domain organization that was not observed with any other Kv channel of known structure. In addition, the structure of Kv3.1 in complex with a known modulator reveals a new ligand pocket. Our work opens avenues toward the understanding of functional specificity of Kv3 channels, and provides a basis for the design of Kv3 modulators with high therapeutic impact.

## Introduction

The characteristic electrical activity of neurons and their ability to conduct, transmit, and receive electric signals, results from the opening and closing of ion channels in the neuron plasma membrane.

Voltage-gated potassium channels (Kv) form a family of transmembrane ion channels which are voltage-sensitive and selective for potassium ions. The Kv3 family consists of Kv3.1 (*KCNC1*), Kv3.2 (*KCNC2*), Kv3.3 (*KCNC3*), and Kv3.4 (*KCNC4*). Kv3.1 channels conduct a delayed rectifying outward current and have a high activation threshold (≈ −20 mV) as well as rapid activation and deactivation kinetics (1). The Kv3.1 channels are, thereby responsible for neuronal repolarization, and high-frequency action potential firing (2, 3).

The Kv3 channels, like other Kv channels, are tetrameric structures of four pore-forming alpha subunits. Each alpha subunit is comprised of six transmembrane helixes (S1–6). The S4 helix has positively charged residues at every third residue, and acts as a voltage sensor that together with S1–3 is denoted the voltage sensing domain (VSD, S1–4). The sensing of voltage triggers a conformational change that opens the channel pore domain (PD, S5–6) and allows a flux of K^+^ ions through the channel (4).

Kv channels often associate with accessory beta subunits like Kvβ1, 2, and 3 (5–8). In some cases, these subunits are involved in N-type inactivation of the channel, as for Kv1.2 regulation by Kvβ1 (5). It was shown that neither Kvβ1 nor Kvβ2 coimmuno-precipitates with Kv3.1 (9) pointing to a distinct regulatory mechanism of the Kv3 family of ion-channels. Other potential accessory subunits such as KCNE1, KCNE2, and KCNE3 were shown to modulate the function of Kv3.1 and Kv3.2 by slowing their activation (10). In the case of KCNE3 it was suggested that it coassembles with Kv3.1 in the rat brain, but not in a universal manner. For example, no association between Kv3.1 and KCNE3 could be observed in rat E18 hippocampal neurons (10). Overall, there is no clear report of Kv3.1 forming a stable complex with a known accessory subunit.

Importance of the Kv3 subclass of potassium channels for CNS-related diseases is supported by identification of several disease mutations connected to rare forms of epilepsy (11, 12). In addition to the high therapeutic potential in seizure treatment, Kv3 modulators might rescue dysfunction and alteration in gamma oscillations. These modulators could regulate diverse cognitive functions such as sensory integration, attention, and working memory (13), which are for example attenuated in Alzheimer's disease (14). Consequently, several Kv3 small molecule modulators with different profiles and properties have been identified. AUT1 (15) and AUT2 (16) have been reported as positive modulators of Kv3.1 and Kv3.2 channels. In addition, H. Lundbeck A/S has discovered a series of Kv3.1 modulators (17) that act as potentiators by lowering the voltage threshold for activation, leading to an increased peak current.

Here, we report the first cryo-EM structures of a Kv3 channel in apo form and in complex with the Lu AG00563 potentiator ligand. Analysis of the structures gives insights into the tetramer association and identifies a novel potentiator binding site of the Kv3.1 channel.

## Results

### Protein biochemistry and apo protein EM structure

Construct design for cryo-EM work was done to ensure optimal relevance of the protein for structure-based drug design. In the case of Kv3.1, two splice isoforms are known, the canonical Kv3.1a isoform and Kv3.1b isoform which has an extension at the C-terminus (18). Both isoforms display identical basal currents but Kv3.1b function is further regulated by a phosphorylation site at the C-terminus (19). Consequently, the selection of the isoform is not critical for structure-based drug design targeting the core channel, independently of the C-terminal end. Expression in HEK293 and purification were performed with the canonical full-length wild type Kv3.1a isoform tetramer referred to as flWT-Kv3.1a. Biochemical analysis of the alpha subunit showed no copurification with any endogenous subunit at a level which could be detected by Coomassie staining. High level of homogeneity and detergent stability were achieved as judged by the size exclusion profile and negative staining analysis of the purified sample (Figure S1, Supplementary Material).

**Fig. 1.**
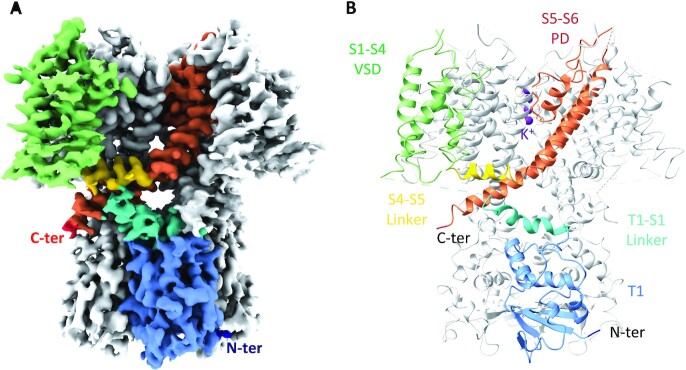
Structure of apo flWT-Kv3.1a. (A) Cryo-EM reconstruction of apo flWT-Kv3.1a. (B) Atomic model of apo flWT-Kv3.1a. The color code is identical in both figures and highlights the different domains and their organization in flWT-Kv3.1a. For clarity, only one monomer has been colored, the remaining three monomers are shown in light gray. The N-terminus end is colored in dark blue, the T1 domain in light blue, the T1-S1 linker in cyan, the VSD in light green, the S4–S5 linker in yellow, the PD in orange, and the C-terminus end in red. Potassium ions visible in the reconstruction were modeled and are colored in purple.

The cryo-EM structure of flWT-Kv3.1a was solved at 2.65 Å overall resolution (Figures S2 and S3, Supplementary Material). Even though the local resolution varied between the PD and the VSDs (Figure S2D, Supplementary Material), the quality of the density, when investigated at different contour level (Figure S4A and B, Supplementary Material), enabled de novo model building (Figure S4, Supplementary Material) of most of the protein (see Materials and Methods). Human flWT-Kv3.1a adopts a tetrameric swapped organization, with the VSD of each subunit interacting with the PD of the neighboring subunit (Fig. 1).

**Fig. 2.**
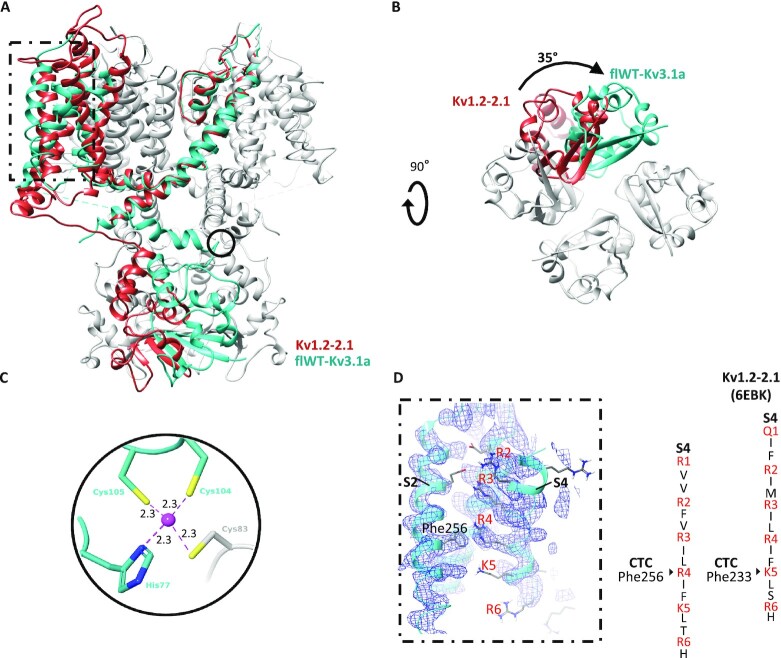
Comparison between the structures of Kv1.2–2.1 and flWT-Kv3.1a. (A) Overlay of a monomer of Kv1.2–2.1 paddle chimera structure (red, pdb code: 6ebk) with a tetramer of flWT-Kv3.1 (cyan, only one monomer colored). The overlay is done on the PD. (B) flWT-Kv3.1a and Kv1.2–2.1 structures are shown from an intracellular viewpoint to illustrate the 35° twisting motion. (C) Coordination site for Zn^2+^ atom (purple, and distances to nearest heavy atom reported in Angstroms) in flWT-Kv3.1a. The Zn-site appears to assist the T1 tetramerization by bridging His77 (T1), Cys104 (T1-S1 linker), and Cys105 (T1-S1 linker) from one monomer, with Cys83 (T1) from a neighboring monomer. (D) Zoom on the VSD. The electron density map and the corresponding atomic model are presented. R2, R3, R4, K5, and R6 residues within the S4 segment are presented in red. In the case of Kv3.1, R4 interacts with Phe256 within the charge transfer center in contrast to the structure of Kv1.2–Kv2.1 showing that R4 is located above Phe233, which interacts with K5. Phe233 in Kv1.2–Kv2.1 is the equivalent of Phe256 in Kv3.1.

Overall, the transmembrane domain organization is similar to what was observed with Kv channels in previously published structures, particularly for Kv1.2 (4, 20–22), which shares the highest sequence similarity with Kv3.1 (RMSD values for different helices or domains are shown in Table S1, Supplementary Material). In contrast, the T1 cytoplasmic domain shows a novel structural feature by adopting a 35° twisted conformation (Fig. 2A and B). Furthermore, the T1 domain tetramerization of Kv3.1 is mediated by a Zn^2+^ atom coordinating interaction between His77, Cys104, and Cys105 from one polypeptide and Cys83 from the neighboring polypeptide (Fig. 2C) similar to the structure of the T1 domain tetramer of Kv4.3 (23).

Another important structural difference with Kv1.2–Kv2.1 could be observed at the VSD. The structure of flWT-Kv3.1a shows that only Arginines R1, R2, and R3 are located above the Phe256 residue (Fig. 2D) within the hydrophobic charge transfer center, which in general consists of a rigid cyclic cap and two negatively charged residues interacting with a positively charged residue in S4, and which facilitates the movement of positively charged residues through the membrane (24). Arginine R4, which is located above the residue Phe233 (equivalent to Phe256 in Kv3.1) in the structure of Kv1.2–Kv2.1, is in the case of Kv3.1 involved in a Π–cation interaction with Phe256 (Fig. 2D). Overall the S4 sensor of Kv3.1, which is characterized by high activation potential, is shifted downward, in comparison to the structure of Kv1.2–Kv2.1. This suggests a more activated state of Kv1.2–Kv2.1.

As observed with other Kv channels, EM density is visible in the center of the selectivity filter (Fig. 1B; Figure S4C, Supplementary Material) corresponding to the average density of distinct K^+^ ions. A total of four sites of potassium ions could be modeled coordinating the residues forming the selectivity filter. Before entering the Kv family-conserved selectivity filter, K^+^ ions pass through the lower gate. The analysis of the pore radius in this region shows that the lower gate of the channel is in an open conformation with a diameter of 3.3 Å (Figure S5, Supplementary Material), similar to other structures solved in the open state, such as Kv1.2 (4, 20–22) and Kv7.1 (25, 26) with similar pore radius at the most constricted region.

### EM Structure with potentiator ligand

**Fig. 3.**
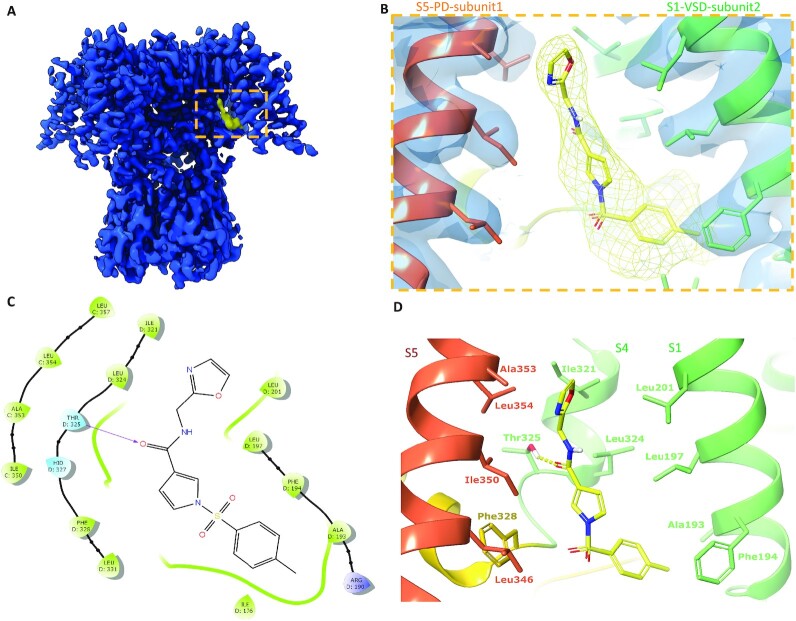
Binding site observed for the Lu  AG00563 positive modulator. (A) Cryo-EM density map (blue) with region of nonprotein density attributed to the ligand (yellow). (B) Close-up view of the ligand binding pocket, displaying both the ligand and protein experimental densities. (C) Simplified diagram of the protein–ligand interactions, highlighting the H-bond with Thr325, hydrophobic residues (green), and polar residues (cyan). (D) Tri-dimensional representation of interactions in the binding site. The S1 and S4 helices are shown in light green, S5 in orange, and the S4–S5 linker in yellow.

H. Lundbeck A/S identified and patented (17) a series of novel compounds which act as Kv3 channel potentiators by shifting the activation threshold to the hyperpolarized direction. Within the series, Lu AG00563 (Ex86 in the patent), showed a good aqueous solubility in our buffer system and could be added to the purified protein at a final concentration of 500 µM without impairing the quality of the sample preparation. The ECΔ5mV parameter corresponds to the effective concentration needed to shift the activation threshold by 5 mV toward the hyperpolarized direction. Measurement of ECΔ5mV was performed with HEK293 cells stably expressing Kv3.1b isoform referred to as flWT-Kv3.1b. The ECΔ5mV value obtained with Lu AG00563 is 2.7 µM (Figure S6, Supplementary Material). Electrophysiology experiments are described in the Supplementary methods. The EM structure with the ligand bound was determined at an overall resolution of 3.0 Å (Figures S7 and S8, Supplementary Material). The organization of the domains and subunits and all the observations made for the apo structure are identical. No variation of the pore radius could be observed and the lower gate does not display any structural rearrangement. After careful analysis of the cryo-EM reconstruction for non protein densities, the density previously observed for the K^+^ ions in the apo-structure were confirmed as well. Furthermore, an additional density, absent in the apo-structure, was observed (Fig. 3A). This density is located at the interface between two monomers where the VSD of one monomer, is interacting with the PD of the neighboring subunit (Fig. 3A and B). Lu AG00563 could be fitted into the density in an unambiguous conformation (Fig. 3B), either manually or assisted by computational docking methods. Analysis of the ligand binding cavity highlights interactions that could be explored for the design of modulators of Kv3 channels (Fig. 3C and D). A favorable geometry for a hydrogen bond interaction is observed between the side chain of Thr325, located within S4 at the VSD–PD interface, and the ligand carbonyl group. Several hydrophobic residues appear to contribute to the ligand interaction. In particular, residues from the S1 helix like Ala193, Phe194, Leu197, Leu201, as well as Leu324 and Phe328 of helix S4, and residues Ile350 and Leu354 of helix S5 from the neighboring monomer.

## Discussion

### Structural differences of Kv3.1 in comparison to other Kv channels

Among the Kv channels for which a structure is available Kv3.1 shares the highest sequence and structure similarity with Kv1.2 (4, 20–22). The major structural difference between Kv1.2 and Kv3.1 is observed at the intracellular T1 domain with a 35° twist between the two T1 domains of both channels. Another major dissimilarity is the zinc-binding site at the base of the T1 tetramerization, which is not observed in Kv1.2. From a functional regulation perspective, Kv1.2 is regulated at the intracellular side by its association via the T1 domain to Kvβ1, which is involved in N-type inactivation of the channel. The lack of confirmation of any association of Kv3.1 with Kvβ1 suggests that the activity regulation of the channel may be performed with distinct mechanisms.

### A novel Kv potentiator-binding site

**Fig. 4.**
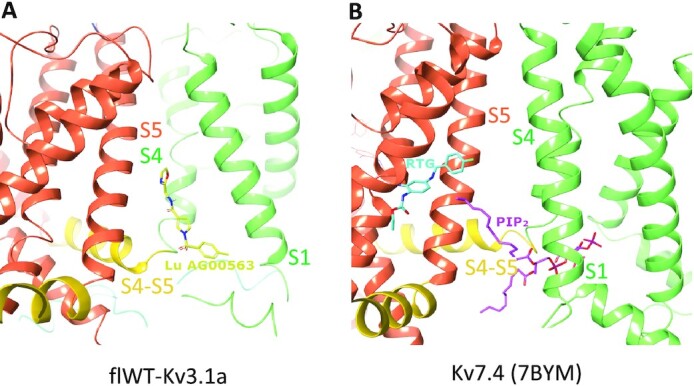
Comparison between the binding site of Lu  AG00563 in flWT-Kv3.1a and the binding sites of PIP_2_ and RTG in Kv7.4. (A) Binding site discovered for Lu AG00563 at the interface between S1, S4, and the S4–S5 linker. The S1 and S4 helices are shown in light green, S5 in orange, and the S4–S5 linker in yellow. Lu AG00563 is shown in yellow. (B) Comparison with the Kv7.4 structure (pdb code: 7bym), highlighting similarities with the PIP_2_ binding site showing interactions with S1, S4, and the S4–S5 linker. In contrast, the RTG binding site is located next to the pore (S5) domain. PIP_2_ is shown in purple and RTG is shown in Cyan.

The binding site for Lu AG00563 at the interface between the VSD and the PD represents a key finding of the present structure. Lu AG00563 displays similar electrophysiological behavior to retigabine (RTG), which is a potentiator inducing a negative shift in the required activation voltage. The structure of RTG bound to Kv7.2 (27, 28) and Kv7.4 (27) was recently solved and shows that the binding site of the compound is located next to the PD between S5 from one subunit and S6 from the neighboring subunit (Fig. 4B). In contrast, Lu AG00563 binds to Kv3.1 between the VSD of one subunit, and the PD of the neighboring subunit (Figs 3 and 4A). Therefore, Lu AG00563 and RTG display similar functional properties by binding to two distinct pockets. Hence, the current structures offer a new insight for how these channels could be modulated by small molecules.

The newly discovered site for Lu AG00563 is located in close proximity to the S4 segment, which is paved with positively charged residues acting as voltage sensors. However, the ligand is not fully entering the S1–S4 bundle. Interestingly, the structure of Kv7.4 bound to PIP_2_ carrying a lipid headgroup entering the S1–S4 bundle (Fig. 4B) shows a related binding site for this endogenous lipid, and has been reported to favor the opening of Kv7 channels (29). The binding site is also a hotspot for several disease related mutations that were reported in the Kv3 class, highlighting the functional importance of the binding site. Some of the mutants occur in the S4 segment, which is strictly conserved within the Kv3 subclass (Figure S9A, Supplementary Material). In particular, point mutations occurring in the VSD that have been found in patients with spinocerebellar ataxia are associated with current amplitude reduction. Some of these mutations involve positive-charge neutralization occurring in S4, such as Kv3.3-R420H (30) or Kv3.1-R320H and Kv3.3-R423H (31). Of particular interest is the T428I mutation observed in Kv3.3 (32), and which is associated with a severe loss of function of the channel (33), since Thr428 is structurally equivalent to Thr325 in Kv3.1 which forms the key hydrogen bond interaction with Lu AG00563 (Figure S9B, Supplementary Material). Taken together, these observations support a role for residues near the Lu AG00563 binding site area in the modulation of the channel activity (Figure S9B, Supplementary Material).

### Impact to drug discovery

The binding site of the Lu AG00563 potentiator at the bottom of S4 and next to the S4–S5 linker has only been previously observed for the nondrug like lipid PIP_2_ in Kv7.4. The hydrogen bond of the carbonyl of Lu AG00563 with the hydroxyl of Thr325 appears to be a key interaction for this binding mode, as it uses the only polar side chain residue present in this intersubunit area between helices S4 and S5. The lack of high-resolution structures for Kv3 channels has previously hindered the development of potentiators by structure-based methods. In particular, Kv3.1 is known as a potential drug target for the treatment of multiple CNS-related disorders. The ion channel structure reported here could open up new opportunities for the design of drug molecules with enhanced properties and offers an excellent starting point to study the drugability of alternative pockets for the discovery and characterization of Kv3.1 modulators.

## Materials and Methods

### Expression of Kv3.1 in HEK293 F cells for cryo-EM work

The cDNA of the wild type full length human Kv3.1 isoform a (flWT-Kv3.1a with uniprot reference P48547) with a carboxy-terminal tag composed of prescission 3C cleavage site followed by GFP was cloned in the expression plasmid pLXBM7, which allows expression of the target protein in mammalian cells with the control of the CMV promoter. The plasmid was produced in large amounts by using the NucleoBond PC 10000 EF kit. HEK293F cells were transfected using PEI 25K linear transfection reagent (Polysciences). Expression was performed at 37°C for 7 hours followed by a second expression phase at 30°C for 72 hours.

### Purification of flWT-Kv3.1a tetramer

To purify the flWT-Kv3.1a tetramer, cells were resuspended in buffer A (Tris 20 mM pH 7.5, KCl 200 mM, MgCl2 1 mM, Lauryl Maltose Neopentyl Glycol 1%, cholesteryl hemisuccinate 0.1%, Protease inhibitor cocktail EDTA free from Roche according to manufacturer recommendations, and DNASE from Roche at 0.01 mg/ml). Cells were homogenized and the flWT-Kv3.1a tetramer was extracted by gentle stirring for 2 hours at 4°C. The nonsoluble fraction was removed by ultracentrifugation at 42,000 rpm at 4°C for 1 hour using Ti45 Beckman rotor. The soluble fraction was mixed with noncommercial Pro-GFP resin (anti-GFP binder coupled to CNBr Activated Sepharose resin from GE-healthcare) pre-equilibrated with buffer B (Tris 20 mM pH 7.5, KCl 200 mM, Lauryl Maltose Neopentyl Glycol 0.01%, and DTT 1 mM). After incubation for 2 hours at 4°C, the resin was washed extensively with buffer B and the flWT-Kv3.1a tetramer was eluted by addition of 100 µl of HRV 3C Protease at 1 U/µl concentration from Takara Bio. Elution was performed overnight at 4°C. Eluted protein was concentrated on vivaspin 100 kDa cutoff Vivaspin Turbo 15 concentrator and injected on a 10/30 Superose 6 size exclusion chromatography column pre-equilibrated with buffer B. Fractions, which correspond to the tetramer were pooled and concentrated to reach 1 mg/ml concentration.

### Computational methods

All modeling was done in Maestro (Schrodinger inc.). Docking of Lu AG00563 was performed with GlideEM (Schrodinger Maestro version 2020–1) in its standard parametrization (Schrödinger Release 2020–1: Glide, Schrödinger, LLC, New York, NY, 2021) assuming a local EM map resolution of 3 Å. The initial sampling phase generated a large number of candidate poses that were then filtered into the five top scoring poses for the refinement phase and visual inspection. Real-space refinement was then performed using the software Phenix and the OPLS3e/VSGB2.1 force field. Following refinement, an unambiguous pose could be selected based on its docking score, the additional electron density explained by the ligand (density score), and visual inspection of chemical interactions between the ligand and the protein environment (34–36).

### Sample preparation and EM analysis

Negative stain electron microscopy showed good size homogeneity, optimal particles distribution, and shape of a typical potassium channel molecule. Subsequently, sample conditions were screened by cryo-EM for the optimal concentration, grids, and freezing parameters. After evaluation of the conditions, quantifoil (2/2) 300-mesh copper grids were glow-discharged for 50 seconds prior to sample freezing. A volume of 3 µl of flWT-Kv3.1a in presence or not of 500 µM Lu AG00563 at a concentration of 2 mg/ml were placed on the grid, blotted for 3.0 seconds and flash frozen in a mixture of liquid propane and liquid ethane cooled with liquid nitrogen using a Vitrobot Mak IV (FEI) operated at 10°C and 100% humidity.

The EM data collection statistics in this study is reported in Table 1. Data were recorded on a FEI Titan Krios microscope operated at 300 kV equipped with a K2 Summit direct electron detector (Gatan Inc.) and Quantum-LS energy filter (slit width 20 eV; Gatan Inc.). The automation of the data collection was done with the software SerialEM (Mastronarde, 2005). Movies were recorded in electron-counting mode fractionating 70 electrons over 40 frames or 60 electrons over 40 frames for, respectively, the flWT-Kv3.1a (apo) or the flWT-Kv3.1a with Lu AG00563 samples. A defocus range of −0.8 to −2.8 µm was used and the physical pixel size was 0.82 Å/pixel. All the movies were gain-normalized, motion-corrected, and dose-weighted with MotionCor2 (37). The micrographs were sorted using FOCUS (38) to clear up those unwell images.

**Table 1.** Data collection parameters and dataset statistics.

Sample	flWT-Kv3.1a apo	flWT-Kv3.1a + 500µM Lu AG00563
Pixel size (Å/px)	0.82	0.82
Dose/frame (e/Å^2^)	1.75	1.5
Number of frames	40	40
Defocus range (µm)	−0.8 to −2.0	−0.8 to −2.0
Movie collected (#)	6’229	5’057
Movie used (#)	3’767	3’509
Particles extracted (#)	1’023’547	623’698
Particles in final reconstruction (#)	362’349	219’839
Final resolution (Å)	2.6	3.0
Resolution range (Å)	2–8	2.2–8.5

### Image processing

The following processing workflows were used for the samples in the study. The aligned movies were imported into CryoSPARC V2 (39, 40). A set of aligned averages with a calculated defocus range of −0.8 to −2.8 μm was selected from which averages with poor CTF estimation statistics were discarded. Automated particle picking in CryoSPARC V2 resulted in 1’023’547 and 623’698 particle locations for respectively, the apo sample and the sample in presence of Lu AG00563. After several rounds of 2D classification, particles were selected and subjected to 3D classification using the multiclass ab initio refinement process and heterogenous refinement. The best resolved classes consisting of 362’349 particles for the apo sample, and 219’839 particles for the Lu AG00563 sample were finally subjected to 3D nonuniform refinement. The overall resolution of the resulted map was estimated at 2.65Å for apo flWT-Kv3.1a and 3.03Å for Lu AG00563-flWT-Kv3.1a based on the Fourier shell correlation (FSC) at 0.143 cutoff (41).

### Model building and refinement

An initial flWT-Kv3.1a was generated using SWISS-MODEL using as templates the Kv1.2 structure (PDB 2a79). Rigid body fitting was initially done in Chimera followed by manually rebuilding of the model in Coot. Residues 120–191, 218–239, 268–280, 295–311, and 372–376 were not modeled due to poor definition of the corresponding densities. Remaining clashes between side chains were detected using Schrodinger version 2019–4, and remodeled using prime (42). Manual inspection of missing H-bonds in the model was used to refine sidechain positions. Finally, real-space refinement was performed in Phenix version 1.17–3644, applying Ramachandran plot restraints (43). PDB reference for flWT-Kv3.1a in apo state is 7PQT. PDB reference for flWT-Kv3.1a in complex with Lu AG00563 is 7PQU. EMDB reference for flWT-Kv3.1a in apo state is EMD-13604. EMDB reference for flWT-Kv3.1a in complex with Lu AG00563 is EMD-13605..

## Funding

The authors declare no funding.

## Authors' Contributions

M.B., M.H., J.K.K., D.R., L.T., and W.A. planned the project. M.B. and W.A. designed the experiments. S.H. and W.A. optimized the protein expression and purification. M.B. and M.C. prepared the cryo-EM grids and collected the data. M.B. performed the cryo-EM data analysis and refinement. R.C. and D.B. built and refined the atomic model. J.K.K. performed the ligand electrophysiological characterization. M.B., D.B., M.H., and W.A. prepared the manuscript. All authors contributed to the finalization of manuscript.

## Supplementary Material

pgac083_Supplemental_File
