# Supplementary material for: Apo and ligand-bound high resolution Cryo-EM structures of the human Kv3.1 channel reveal a novel binding site for positive modulators
Source: PNAS Nexus. 2022 Jun 16;1(3):pgac083. doi: 10.1093/pnasnexus/pgac083 (PMC9896932; doi:10.1093/pnasnexus/pgac083)
Supplement: pgac083_Supplemental_File [file pgac083_supplemental_file.pdf]

Supplementary Information for

Apo and ligand-bound high resolution Cryo-EM structures of the human Kv3.1 channel reveal a novel binding site for positive modulators

Mathieu Botte[1], Sophie Huber[1], Denis Bucher[1], Julie K. Klint[2], David Rodríguez[2], Lena Tagmose[2], Mohamed Chami[3], Robert Cheng[1], Michael Hennig[1] and Wassim Abdul Rahman[1*]
[1] leadXpro AG, PARK InnovAARE, 5234 Villigen, Switzerland
[2] H. Lundbeck A/S. Ottiliavej 9, 2500 Valby, Denmark
[3] BioEM laboratory, Biozentrum, University of Basel, Basel, Switzerland

Corresponding author: Wassim Abdul Rahman
Email: wassimabdulrahmanresearch@gmail.com

This PDF file includes:

Extended methods
Figures S1 to S9
Table S1

Extended Methods

Kv3.1b electrophysiology using automated patch clamp (QPatch)

Patch-clamp recordings were performed using the automated recording system QPatch-16x/QPatchII-48x (Sophion Bioscience, Denmark). HEK-293 cells stably expressing human flWT-Kv3.1b was used for the experiments. On the day of the experiment the cells were detached by Detachin and resuspended in serum free medium containing 25 mM HEPES and 100U/ml Penicillin/Streptomycin. For each experiment cells were centrifuged, media removed and the cells were resuspended in extracellular buffer containing (in mM): 145 NaCl, 4 KCl, 1 MgCl2, 2 CaCl2, 10 HEPES and 10 glucose; pH 7.4 adjusted with NaOH, 305 mOsm. Single cell whole-cell recordings were carried out using an intracellular solution containing (in mM): 120 KCl, 32.25/10 KOH/EGTA, 5.374 $CaCl_2$, 1.75 $MgCl_2$, 10 HEPES, 4 Na_2ATP, pH 7.2 adjusted with KOH, 295 mOsm. Cell membrane potentials were held at -80 mV and currents were evoked by voltage steps (50 ms duration) from -70 mV to +10 mV (in 10 mV increments). Vehicle (0.33% DMSO) or increasing concentration of compound were applied and the voltage protocol was run 3 times/concentration (resulting in 3 min cpd incubation time). Five increasing concentrations of compound were applied to each cell. Leak subtraction protocol was applied at -33% of the sweep amplitude, and serial resistance values were constantly monitored. Any cell where serial resistance exceeded 25 MOhm, membrane resistance less than 200 MOhm or current size at -10 mV less than 200 pA was eliminated from the subsequent analysis.

Data analysis was performed using Sophion's QPatch analyzer software in combination with Microsoft Excel™ (Redmond, WA,USA). Current voltage relationships were plotted from the peak current at the individual voltage steps normalized to the vehicle addition at 10 mV. The voltage threshold for channel activation was defined as 5% activation of the peak current at 10 mV in presence of vehicle. The activity of the compounds was described as the ability to shift this current voltage relationship to more hyperpolarized potentials and is given as the maximum absolute shift possible at the tested concentrations (0.37, 1.11, 3.33, 10, 30 µM). Concentration response curves were plotted from the threshold shift at the individual concentrations and were fitted excel fit model 205 sigmodal dose-response model (fit=A+((B-A)/1+((C/x)^D)))), where A is the minimum value, B the maximum value, C the EC50 value and D the slope of the curve. The concentration needed to shift the threshold 5 mV was readout from this curve ($EC_{\Delta 5mV}$).

Figure S1

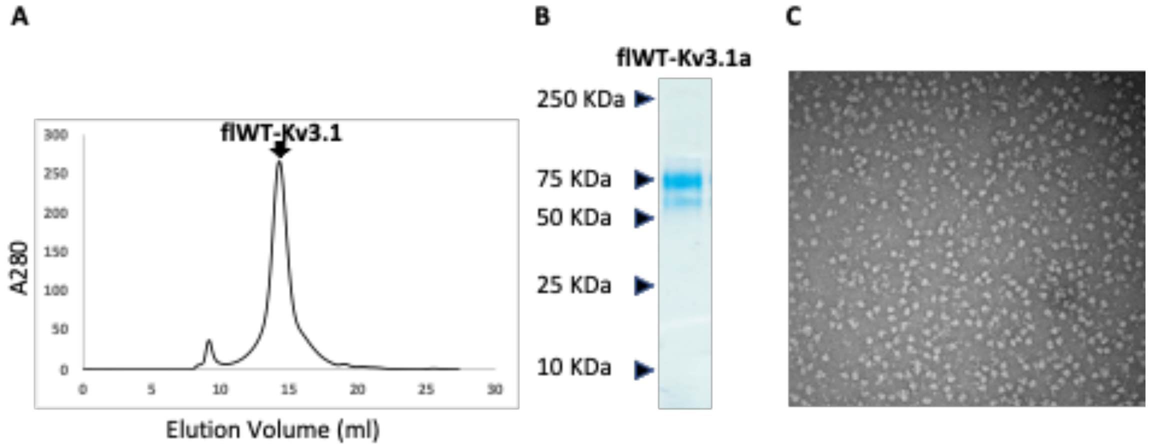

Purification and negative staining analysis of flWT-Kv3.1a tetramer.

A. After affinity purification the protein sample was subjected to superose 6 Size Exclusion chromatography. The peak corresponding to the tetramer is indicated with an arrow. **B.** The final sample was analyzed by SDS-PAGE. Positions of the protein markers bands are indicated with arrows on the left side. **C.** The final protein sample was analyzed by negative staining.

Figure S2

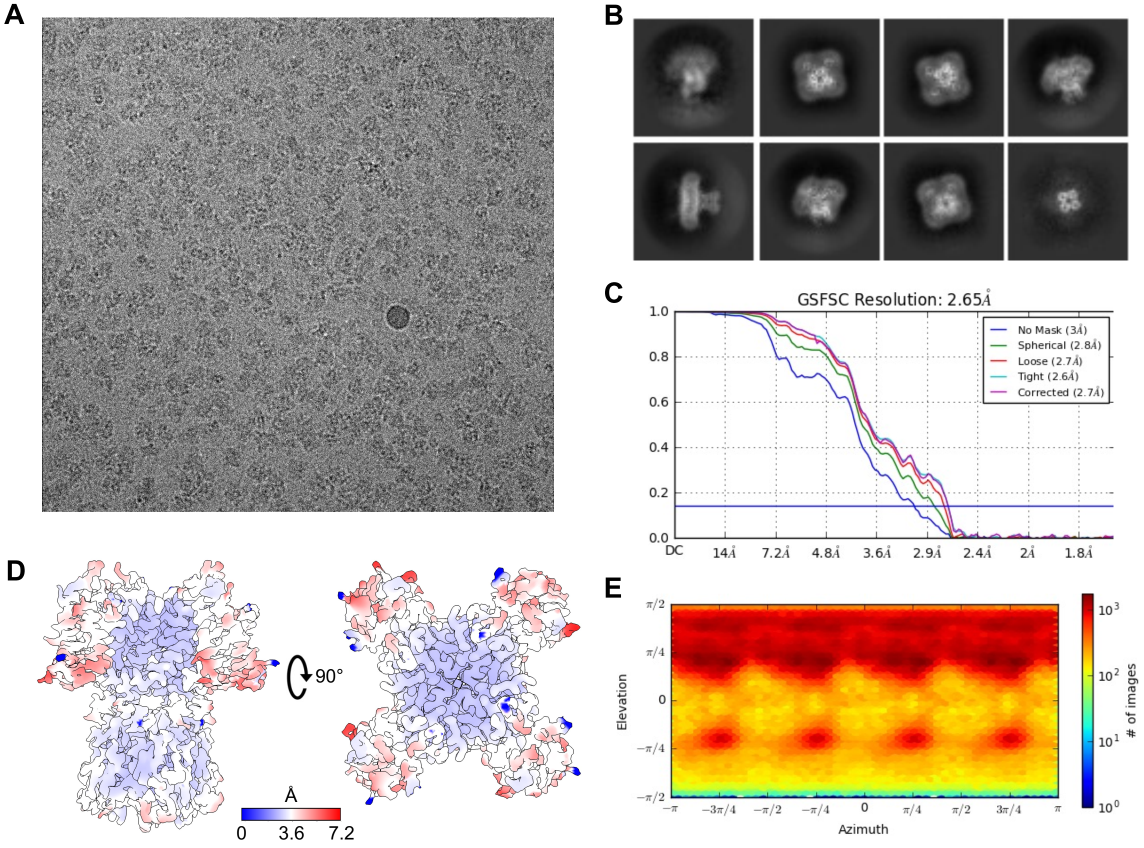

Cryo-EM acquisition and map of apo flWT-Kv3.1a

A. Example of a micrograph of vitrified apo flWT-Kv3.1a. **B**. Example of 2D class averages. **C.** Gold standard Fourier-Shell correlation resolution plot with a cut-off at 0.143 indicated by a blue line. **D.** Map of the apo flWT-Kv3.1a with color-coded local resolution. **E.** Angular distribution represented as a heat map showing the number of particles for each viewing angles.

Figure S3

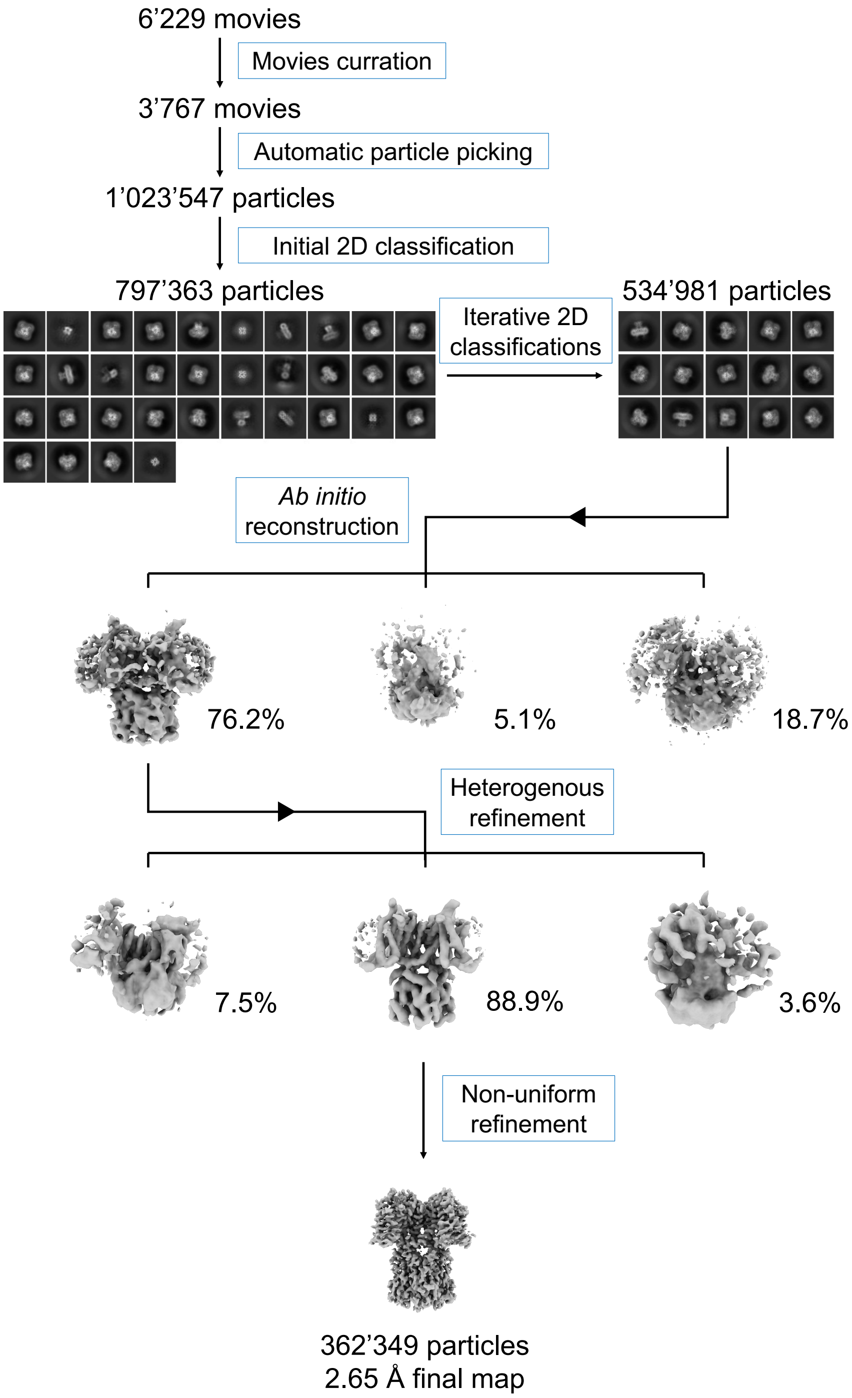

Cryo-EM processing scheme of apo flWT-Kv3.1a.

Graphical description of the processing scheme applied to the apo flWT-Kv3.1a dataset. Full description can be found in the “Materials and Methods” section.

Figure S4

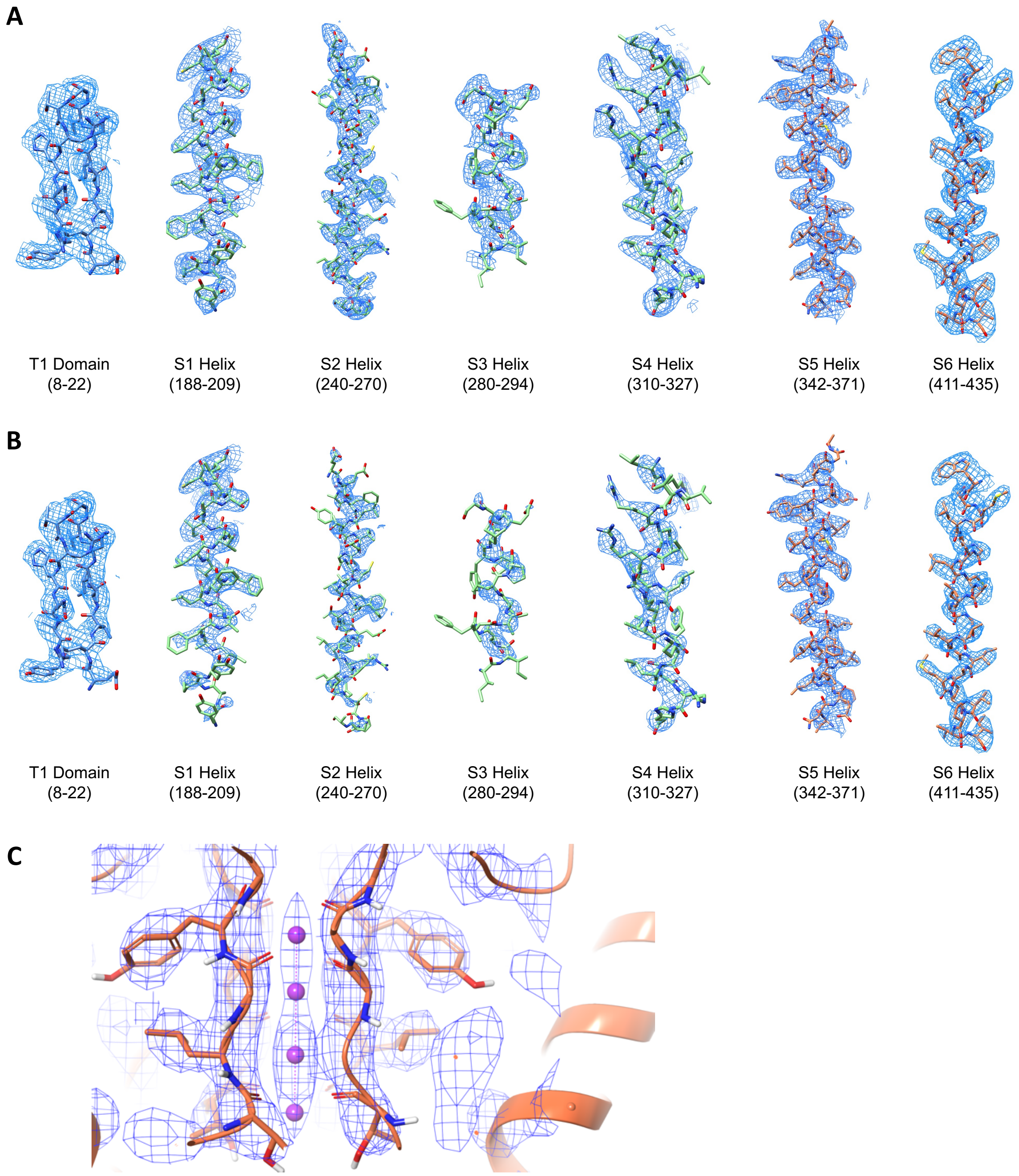

Agreement of the flWT-Kv3.1a Cryo-EM map and the corresponding model.

A-B. Regions of flWT-Kv3.1a shown with the cryo-EM map contoured at 0.4 (A) and 0.6 (B) with the model superimposed. Density map is colored in light blue and the models are colored similarly to Figure 1. **C.** Potassium ions visible in the selectivity filter were modeled and are colored in purple.

Figure S5

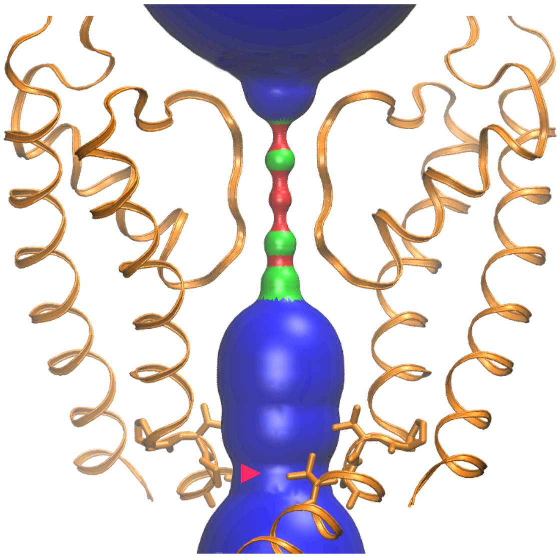

Cartoon representation of the cylindrical radius within the pore

The pore radius is shown as calculated with the HOLE2 program, indicating an open channel at the lower gate region indicated with a red arrow.

Figure S6

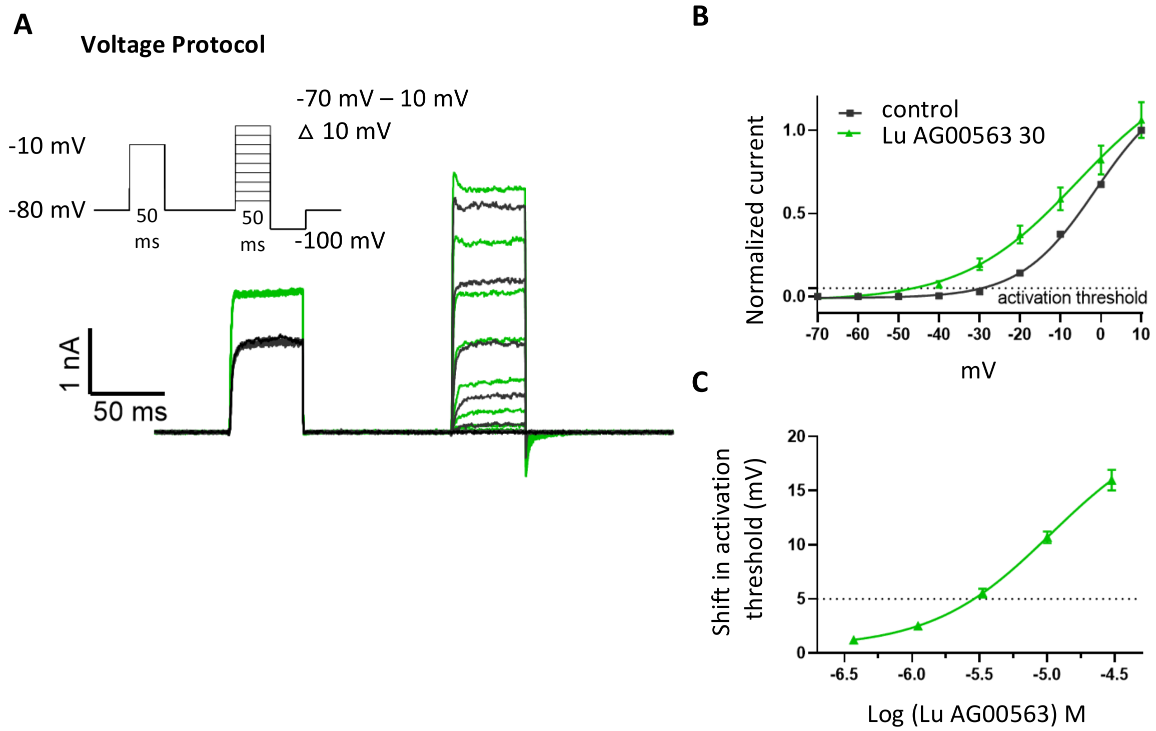

Characterization of Lu AG00563 on flWT-Kv3.1b channel

A. Representative traces for HEK293 cells stably expressing flWT-Kv3.1b as measured by automated electrophysiology using QPatch. Cells were subjected to a 50 ms step to -10 mV followed by 100 ms recovery before an IV protocol was applied from -70 mV to 10 mV in 10 mV increments (voltage protocol insert). Black current traces are baseline, where green is in presence of 30 µM Lu AG00563. **B.** Current-voltage relationships of flWT-Kv3.1b at baseline (black) and in presence of 30 µM AG00563 (green). Dashed line indicates the definition of the activation threshold. **C.** Concentration dependent hyperpolarizing shift in activation threshold by Lu AG00563 resulting in an ECΔ5mV of 2.7 µM. Dashed line indicates 5 mV potency measure point. All results are based on 5 individual recordings and the DMSO concentration is kept constant throughout the experiments.

Figure S7

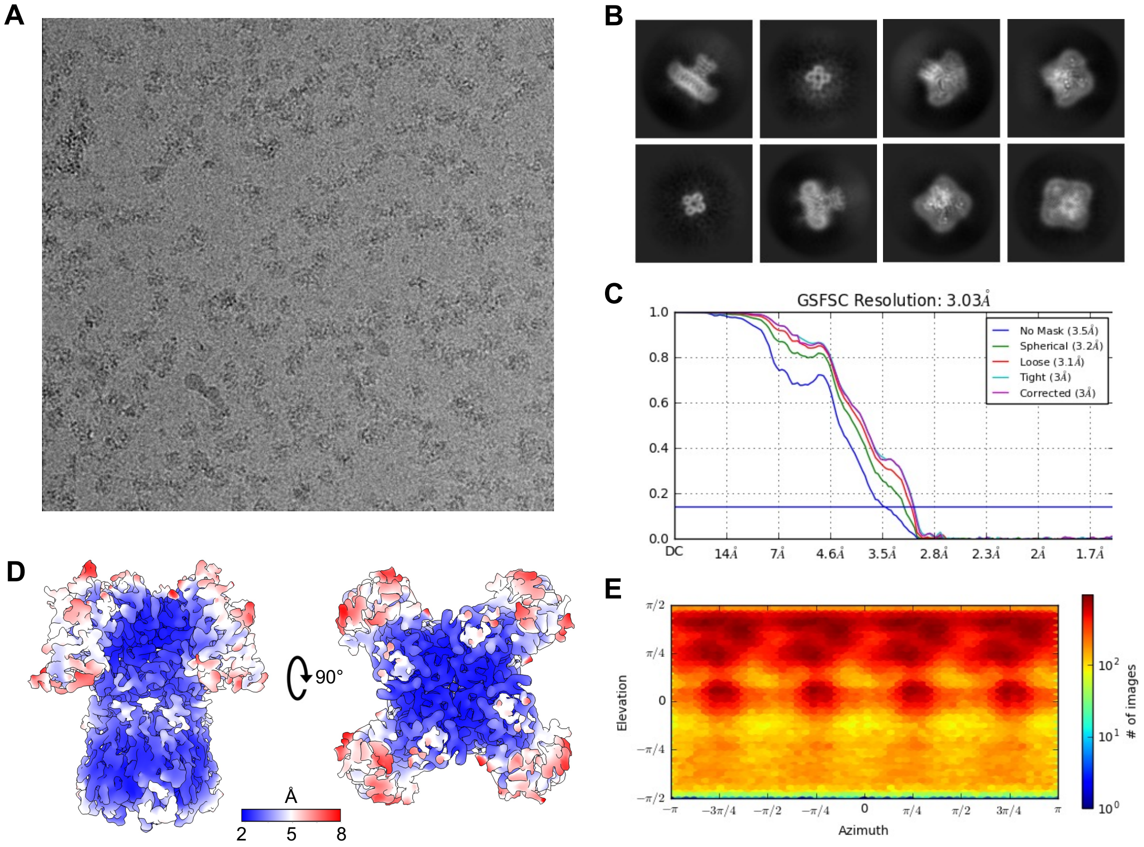

Cryo-EM acquisition and map of flWT-Kv3.1a in presence of Lu AG00563

A. Example micrograph of vitrified flWT-Kv3.1a in presence of Lu AG00563. **B.** Example of 2D class averages. **C.** Gold standard Fourier-Shell correlation resolution plot with a cut-off at 0.143 indicated by a blue line. **D.** Map of the flWT-Kv3.1a in presence of Lu AG00563 with color-coded local resolution. **E.** Angular distribution represented as a heat map showing the number of particles for each viewing angles.

Figure S8

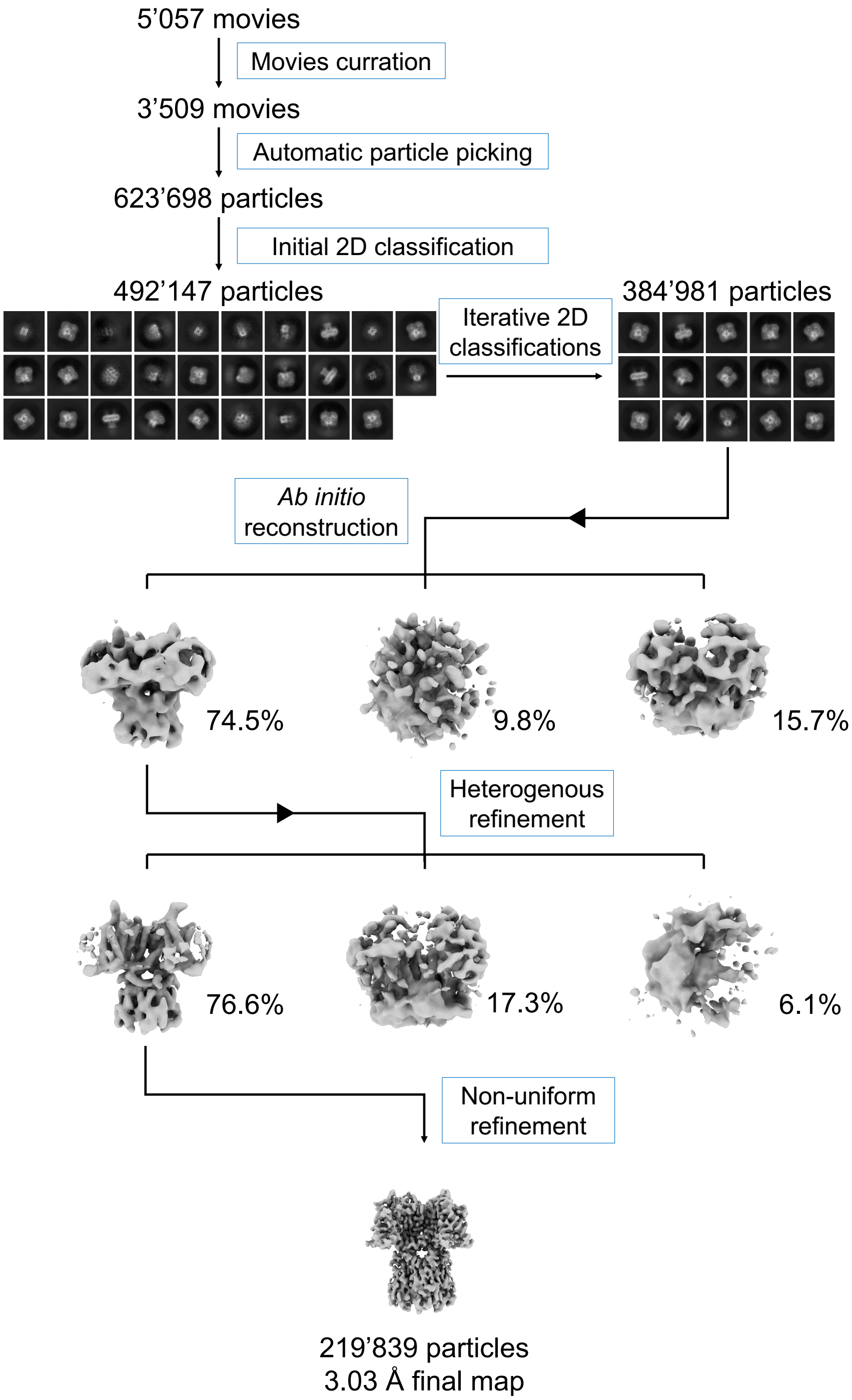

Cryo-EM processing scheme of flWT-Kv3.1a in presence of Lu AG00563.

Graphical description of the processing scheme applied to the flWT-Kv3.1a in presence of Lu AG00563 dataset. Full description can be found in the “Materials and Methods” section.

Figure S9

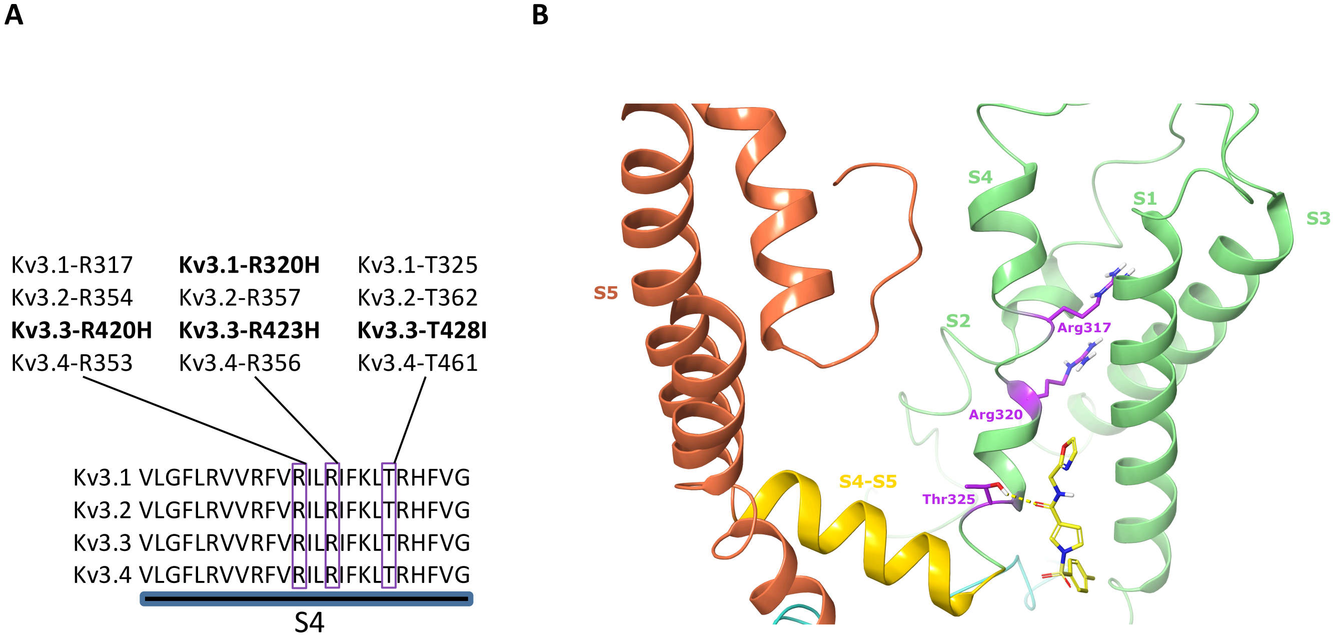

Region of disease related mutations in S4 in the VSD that were reported in the Kv3 class.

A. Sequence alignment of human Kv3.1, Kv3.2 and Kv3.3, showing a high degree of conservation in the sequence of S4 in the VSD. Reported disease related mutations occurring in a class member are shown in bold. The corresponding residue in another class member is indicated **B.** Representation of the disease relevant Arg317, Arg320, and Thr325 residues (purple) in S4 within the VSD (light green), in the vicinity of the Lu AG00563 ligand pocket.

Table S1

Helice or domain compared between the flWT-Kv3.1a structure and the Kv1.2-2.1 structure (PDB 6EBK)	RMSD in Å
S1*	2.14
S2*	4.04
S3*	3.45
S4*	3.38
S5*	0.71
S6*	0.69
Selectivity filter *	0.60
Pore domain (S5 + S6) *	1.6
T1 domain *	Can not be calculated due to domain rotation
T1 domain **	2.357

RMSD values resulting from comparison between the flWT-Kv3.1a structure and the Kv1.2-2.1 structure (PDB 6EBK).

** The alignment was performed based on the trans-membrane region*
*** The alignment was performed based on the T1 domain*
